# Exploring the link between Paradoxical leadership and nurses’ career maturity: the mediating role of organizational learning

**DOI:** 10.1186/s12912-025-03179-6

**Published:** 2025-05-19

**Authors:** Amal Diab Ghanem Atalla, Wafaa Hassan Mostafa, Mohamed Saad Saleh Ali

**Affiliations:** 1 Department of Nursing Administration, Faculty of Nursing, Alexandria University, Alexandria, Egypt; 2 Department of Nursing Administration, Faculty of Nursing, Damanhour University, El-Behira, Egypt

**Keywords:** Paradoxical leadership, Nurses, Career maturity, Organizational learning, Mediating factor

## Abstract

**Background:**

Paradoxical leadership, which strikes a balance between control and flexibility, is becoming more and more acknowledged as being essential for promoting nurses’ career maturity, adaptation, and continuous development in today’s complicated healthcare environments. Nevertheless, little is known about the processes that connect leadership traits to professional development, especially the part played by organizational learning.

**Aim:**

This study aimed to evaluate nurses’ professional maturity and perceived paradoxical leadership, to investigate their relationship, and to determine the mediating role of organizational learning in this relationship.

**Design:**

Following STROBE criteria, a descriptive cross-sectional design was employed.

**Methods and tools:**

A descriptive cross-sectional study was carried out at a general hospital in Egypt with 380 nurses. Participants filled out the Organizational Learning Scale, the Career Maturity Questionnaire, and the Paradoxical Leadership Scale (PLS). Following STROBE principles, data were analyzed using ANOVA, Pearson correlation, and structural equation modeling (SEM).

**Results:**

High degrees of organizational learning, professional maturity, and paradoxical leadership were noted by nurses. All of the factors showed significant positive relationships with one another. Both career maturity (β = 0.144, *p* = 0.004) and organizational learning (β = 0.136, *p* = 0.007) were directly impacted by paradoxical leadership. Additionally, organizational learning moderated the association between leadership and career maturity (indirect effect = 0.0245; total effect = 0.756) and had a direct influence on career maturity (β = 0.180, *p* < 0.001). An excellent fit was shown by the model fit indices (CFI = 1.000, IFI = 1.000, RMSEA = 0.073).

**Conclusion:**

Nurses’ career maturity is greatly increased by paradoxical leadership, with organizational learning serving as a crucial mediating factor. Enhancing these elements through learning programs and leadership development can benefit nurses’ career development and patient outcomes.

**Clinical trial number:**

Not relevant.

## Background

The current competitive environment of healthcare organizations necessitates the involvement of leaders who can effectively manage tensions and easily handle the conflicting and contradicting demands of these complex environments. Paradoxical leadership has emerged as a result of the strong internal struggle and the growing complexity of the external environment [1, 2]. The term “paradoxical leadership” was originally used by Zhang et al. (2015), who defined it as managers’ use of linked but conflicting behaviors to satisfy organizational structures and employees’ tasks [3]. According to a recent World Health Organization [WHO] and International Labour Organization [ILO] study from 2022, work is now more than ever seen as a risk and an opportunity for employees’ maturity [4]. Paradoxical leaders are capable of identifying and redefining job demands or dangers in a more flexible manner, as well as productive opportunities or job resources [5]. The World Health Organization’s “State of the World’s Nursing 2020” report, which promotes funding for nursing education and leadership, has a significant impact on global health policy, and the report’s ultimate goals are to advance critical competencies, strengthen the nursing profession, and shape nursing care paradigms to develop nursing and improve access to healthcare globally [6].

Stronger leadership and ongoing professional development are further promoted as global policy priorities in the WHO’s State of the World’s Nursing 2020 report and the Global Strategic Directions for Nursing and Midwifery (2021–2025), which emphasize the value of organizational learning as a tool for skill development and adaptation. In a similar vein, the ICN’s 2023 report Recover to Rebuild highlights the importance of investing in nursing leadership to transform healthcare delivery and strengthen system resilience. These objectives are closely related to improving career maturity through adaptive leadership and structured learning. The emphasis and applicability of the current study are justified by these international directives, which emphasize the pressing need to look into how learning settings and leadership styles interact to enhance nurses’ professional development [7]. The growing awareness of work as a risk and a chance for professional growth for healthcare workers, especially nurses, is highlighted by recent international reports from the World Health Organization (WHO), International Labour Organization (ILO), and International Council of Nurses (ICN). Depending on how they are handled, changing workplace expectations can either impede or foster professional maturity, according to the WHO and ILO (2022). This is in line with the idea of paradoxical leadership, which allows leaders to reinterpret and manage job expectations by striking a balance between empowerment and control, turning possible stressors into opportunities for growth [4–8].

Nurses must constantly adapt, make important decisions, and advance their careers in the complicated and quickly changing healthcare environment, especially in situations with high patient loads, resource limitations, and changing clinical demands. By promoting both stability and autonomy in their jobs, paradoxical leadership, which strikes a balance between seemingly incompatible behaviors like control and empowerment or structure and flexibility, is thought to be essential for developing nurses’ professional maturity. Key components of professional development, such as critical thinking, resilience, and role conflicts, are all facilitated by this leadership style for nurses. It is suggested that organizational learning serves as the vehicle that makes this relationship possible by fostering a culture of information exchange, skill development, and continual improvement. Organizational learning serves as a bridge that converts leadership behaviors into long-term professional growth by empowering nurses to learn from experience, collaborate efficiently, and adjust to new challenges. This makes it especially pertinent in high-pressure healthcare settings like the one under study.

## Literature review

### Paradoxical leadership

When it comes to managing superior-subordinate relationships, decision-making, power dynamics, and establishing a work environment, leading paradoxically means acting in a way that balances contradictions and close ties. This approach seems competitive, but it is interrelated and meets competing demands at work, based on the twin meanings of meeting the organizational structure’s needs and the subordinates’ specific needs [9]. Zhang et al. (2015) suggested the following five characteristics of paradoxical leadership to describe “both-and” traits: (1) Blending self-centredness and other-centeredness: the ability of a leader to maintain their position as the main impact on the workplace while also acknowledging the needs of their team members and allowing followers to take on leadership roles. (2) Preserving both closeness and distance: the ability to preserve hierarchical boundaries when resolving work-related issues and fostering close relationships with subordinates. (3) Allowing for individualization while maintaining a uniform approach to subordinates: maintaining a balance between consistency and uniqueness by treating followers equally according to agreements and regulations while also creating exceptions depending on each person’s preferences and skills. (4) Enforcing work requirements while permitting flexibility: the capacity to exercise behavioral control (via work processes, for example) while permitting flexibility. (5) Sustaining autonomy while retaining decision control: the capacity to concurrently foster subordinate autonomy and preserve decision control (as in output control) [3, 10].

Gains in organizational performance, creativity, competitiveness, and success provide as evidence that paradoxical leadership is a successful leadership style for managing the complex environments that modern firms face. It also positively affects nurses’ commitment, work engagement, attitudes about their professions, and exceptional performance. Paradoxically, leading also gives management support that fosters nurses’ inventive and creative behaviors, learning opportunities, and career progression prospects. It also shows that nurses are being offered more opportunities to advance their careers and grow professionally. Paradoxical leadership promotes nurses’ professional development in several significant ways. By finding a balance between control and flexibility, it promotes decision-making autonomy, empowering nurses to take charge and develop their critical thinking skills. Encouraging open communication, taking measured chances, and lifelong learning without fear of consequences, it also promotes psychological safety. Furthermore, this leadership approach promotes adaptability, which helps nurses effectively handle complex and evolving healthcare scenarios. As a result, nurses gain professionalism, competence, and confidence, all of which contribute to their overall career maturity [3, 11].

Akeel and Abd ElFattah’s “Paradoxical Leadership and Its Effect on Burnout among Staff Nurses” study sought to determine how staff nurses perceived paradoxical leadership practices, how burned out they were, and how perceived paradoxical leadership affected nurses’ burnout. The results showed that only 16.2% of the nurses reported having a positive opinion of paradoxical leadership, whereas over half (53%) had a negative opinion. Furthermore, only 3.1% of the nurses reported severe degrees of burnout, compared to over three-quarters (77.7%) who reported mild levels. Burnout levels among staff nurses were found to be significantly correlated with their opinions of paradoxical leadership. According to the study’s findings, nursing staff burnout may be decreased by strengthening paradoxical leadership characteristics [12].

### Career maturity

One of the most essential variables in encouraging nurses’ future professional development is career maturity. It has a big impact on their decision-making, professional growth, job and career satisfaction, and retention in the modern workplace [13]. It is defined as a person’s readiness to plan and make appropriate career decisions, including knowing how to go about making these decisions and how reasonable and stable one’s professional decisions are over time [14, 15]. Four aspects of career maturity are as follows: Career exploration is defined as “Investigating various career options and what they entail; placing self-abilities following career, interests, and work goals so that they can be better adjusted to careers in the future”. Career planning refers to “developing both short- and long-term goals for the career development within a specific timeline for accomplishing these goals”. Vocational self-concept comprises “expressing self-concept, or understanding of self, which evolves, seeking career satisfaction through work roles in which individuals can express themselves and further implement and develop their self-concept. Making a career choice involves “collecting the needed information, weighing the costs and benefits of the choices, and making final decisions that fit the personal characteristics and overall life goals [16, 17].

Higher professional maturity among nurses is associated with greater career accomplishments, more age-appropriate job choices, and preparation for careers outside of nursing. Furthermore, there is a substantial linkage between career maturity and future vision, as evidenced by the large positive relationship that high career maturity has with future time perspective. Furthermore, nurses’ readiness in their career decision-making process is greatly aided by career maturity, underscoring its significance in attaining professional success. All things considered, nurses with high career maturity are more equipped to recognize occupational preferences, make mature and realistic career decisions, and efficiently plan and prepare for their intended careers [18]. Organizational learning culture is a main factor for career maturity, where it is an antecedent of career development through which organizations adopt various developmental activities. While leadership styles greatly increase learning, organizational learning mediates greatly between models of leadership, and the enhancement of career maturity and development through involving various career developmental activities to accomplish peak organizational goals [19, 20].

### Organizational learning

Organizational learning has been the center of attention for the last two decades [21]. It is a process that involves intuition, interpretation, integration, and institutionalization at the individual, group, and organizational levels. It is necessary to actively acquire, build, and incorporate knowledge to generate the organizational resources and abilities needed to improve organizational performance [22]. The learning process in healthcare organizations is dynamic and integrative, emphasizing how learning is connected to the events and their surrounding context [23]. The organization’s conversion entails accepting the mission of the learning organization and cultivating an atmosphere that views the company’s programs as outstanding learning opportunities and values learning from both triumphs and mistakes. Supporting employees in their learning process, hearing their viewpoints, and focusing on their training and ability to learn from their job are the five primary components that make up organizational learning. Decentralization and authority delegation, staff training and development support, and idea sharing are all components of employee empowerment. Knowledge management includes tracking employee expertise and capacity for creative thinking, identifying best practices, new data, and operational procedures, as well as developing learning process strategies. The term “application of technology” refers to the use of sophisticated information systems to deliver information quickly and readily, as well as the teaching and learning process that makes use of these technologies [24, 25].

Organizational learning provides the framework for complex, interconnected, dynamic systems in the health care system, where each operational unit must learn and perform its assigned tasks to improve patient safety and minimize errors. To improve patient and organizational results, healthcare professionals are expected to participate in ongoing education to stay up to date on the latest knowledge and skills [26]. Organizational learning leads to technological innovation, process optimization, and product enhancement, which raises competitiveness and promotes long-term organizational success and expansion. Without organizational learning, the organization will stagnate and be unable to adapt to changes in its surroundings [27].

### Theoretical framework

Career theory sheds light on how individuals make decisions, set goals, and perform differently concerning their academic and professional objectives. Career theories offer frameworks for comprehending how different factors interact to influence a person’s career possibilities and advancement throughout their lifetime. According to Bandura’s general social theory (1986) [28] is the foundation of the comprehensive Social Cognitive Career Theory (SCCT). The comprehensive theory of SCCT emphasizes the role that particular cognitive characteristics play in careers. This demonstrates the complex interactions that take place between individuals, their surroundings, and their actions. Self-efficacy and social context are said to influence behavior. According to SCCT, people are capable of a certain amount of self-direction or organization, but they also deal with several issues that might either support or contradict personal organization.

According to organizational learning theory, knowledge sharing, information acquisition, and shared insights let organizations continuously adapt and get better. This learning process strengthens career maturity in nursing by improving professional growth, decision-making, and problem-solving skills. By promoting both adaptability and conformity to norms, paradoxical leadership creates a learning culture that empowers nurses to successfully incorporate new information. By fostering critical thinking and adaptation, organizational learning acts as a mediator, converting leadership influences into real advantages for career growth. This dynamic strategy guarantees that nurses’ leadership-driven learning results in increased professional maturity [29].

**Fig. 1:**
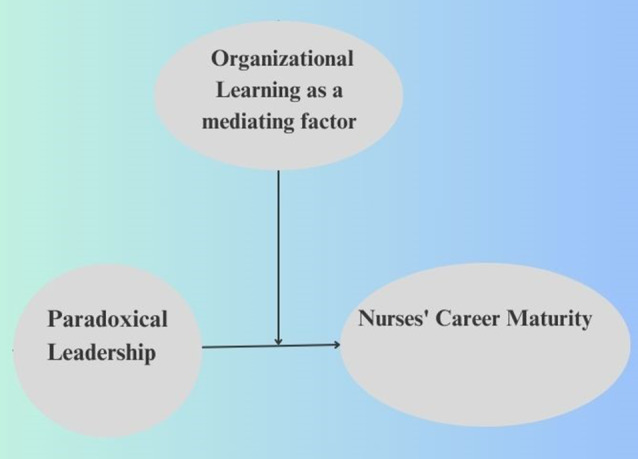
The conceptual framework of the study as recommended by the researchers

The researchers developed a conceptual model for this investigation based on the previously indicated conceptualizations (Fig. 1). The following conceptual framework is hypothesized, assuming that organizational learning serves as a mediating factor, nurses’ career maturity is the dependent variable, and paradoxical leadership is the independent variable:

### Significance of the study

Compared to other leadership styles like transformational or servant leadership, paradoxical leadership is especially applicable in the nursing profession since it successfully manages the conflicts and complexities that are a part of healthcare environments. Paradoxical leadership strikes a balance between seemingly incompatible demands, such as control and flexibility, autonomy and oversight, or structure and adaptability, enabling nurses to navigate unpredictable clinical environments while maintaining high performance [1, 2]. This contrasts with transformational leadership, which primarily focuses on vision and motivation, or servant leadership, which prioritizes employee well-being. In hospitals, where nurses must simultaneously follow stringent rules while using critical thinking and flexibility in patient care, this balance is especially important. Paradoxical leadership is particularly well-suited to the dynamic and high-stakes character of the nursing profession because it fosters both stability and creativity, allowing nurses to grow as professionals via resilience, confidence in their abilities to make decisions, and ongoing learning.

This study is imperative because it advances our knowledge of how paradoxical leadership, which balances seemingly incompatible leadership traits like autonomy and control, may effectively foster career advancement in demanding, fast-paced fields like nursing. The study offers insights into how ongoing knowledge-sharing and flexible learning environments can improve career maturity in a variety of healthcare settings by looking at the mediating effect of organizational learning. The research is pertinent well beyond the particular context of Egyptian hospitals because these findings are generally applicable to international initiatives aimed at enhancing workforce development, leadership effectiveness, and professional growth in healthcare systems dealing with rapid change, resource constraints, and growing complexity.

This study fills important theoretical and practical gaps in the body of knowledge about nursing leadership and career development. Although servant and transformational leadership place a strong emphasis on encouragement and support, they frequently overlook the vital balance between flexibility and control, leading to divergent views on how to effectively lead in hospital settings that are both dynamic and structured. A viable substitute is paradoxical leadership, which combines opposed traits like discipline and empowerment [8]. This is particularly true when combined with organizational learning, which is essential for promoting ongoing skill development and career advancement. Given the critical role nurses play in providing high-quality healthcare and the difficulties they have when adjusting to demanding, complicated environments, it is imperative to comprehend how paradoxical leadership affects their professional development. To influence leadership strategies, training initiatives, and policy creation that improve nurses’ decision-making, resilience, and long-term professional growth, this study intends to investigate this relationship, with organizational learning acting as a mediating component.

### The research questions are

- What are the organizational learning, professional maturity, and paradoxical leadership levels of nurses?
- Is there a connection between nurses’ organizational learning, career maturity, and paradoxical leadership?
- Does organizational learning **mediate** the relationship between nurses’ career maturity and paradoxical leadership?

### Study design

A descriptive correlational cross-sectional study design was employed in an Egyptian hospital with adherence to the STROBE (Strengthening the Reporting of Observational studies in Epidemiology) guidelines.

### Setting

The Ministry of Health and Population (MOHP)-affiliated Itay El Baroud General Hospital, which has 220 beds, served as the study site. All medical and surgical inpatient care units (*n* = 16) as well as intensive care units (ICUs) were included. Important actions were taken by this institution to meet the General Authority for Health Accreditation and Regulation’s (GAHAR) patient safety requirements. It is one of the biggest hospitals in Egypt and the Middle East, with a vast patient base and a wide geographic reach. It acts as a tertiary referral center for many hospitals in the nation as well as those in adjacent countries. The community as a whole benefits from improved patient outcomes and higher-quality healthcare services that can be achieved through improved career planning and retention for nurses. Because of its vibrant and diversified nursing staff, the hospital was chosen for the study. This creates a rich context for researching the connection between career development and paradoxical leadership. The hospital is known for its commitment to organizational learning and professional growth, making it the ideal location to study how these factors mediate; the study’s goals align with the hospital’s ongoing efforts to improve nurse care and leadership practices; and the active and diverse nursing staff makes it possible to fully understand how leadership philosophies impact nursing performance and career advancement.

### Sampling

Purposive sampling is a sort of non-probability sampling that was used in this investigation. Participants were specifically chosen based on predetermined inclusion criteria that were pertinent to the goals of the study. This strategy made sure that only nurses who had the necessary training and exposure to the hospital setting were included. Although the phrase “convenience sampling” was once used, it would be more accurate to refer to the method as purposive because the selection of participants was based on particular traits rather than just accessibility.

Registered nurses who provided direct patient care in pre-selected hospital units were part of the target demographic. Participants had to meet three requirements to be eligible: (1) be present throughout the data collection time; (2) be actively involved in patient care; and (3) have at least one year of experience in their current unit to guarantee familiarity with hospital regulations and processes. Newly hired employees, nurses on leave, and nurses in administrative or non-clinical duties were all automatically eliminated under these criteria.

**Fig. 2:**
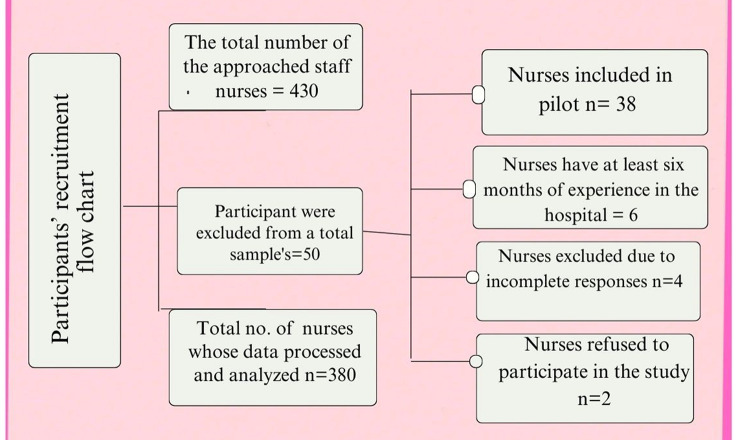
Participants’ recruitment flow chart

A sample size calculation was conducted using Epi Info™ (Version 7.2). The minimum required sample size was determined to be 203 participants based on the cross-sectional design of the study, the total estimated population of eligible nurses (*N* ≈ 430), a 95% confidence level, a 5% margin of error, and assuming a 50% expected frequency to maximize sample size estimation (since no prior data on the prevalence of paradoxical leadership or career maturity was available). However, a larger sample of 380 nurses was gathered to improve generalizability, allow possible subgroup analyses (e.g., by unit type, experience level), and increase the study’s statistical power. This method accounts for the variability brought forth by sociodemographic and work-related factors while guaranteeing strong representation of the eligible nursing population. (The Participants’ recruitment flow chart is illustrated in Fig. 2.)

### Study tools

#### Sociodemographic characteristics section

The researchers questioned the age, gender, years of work unit, education, and nursing experience of the study participants.

#### Paradoxical leadership scale (PLS)

Its goal was to gauge nurses’ perceptions of paradoxical leadership. The scale was designed by Zhang et al. (2015) [3] and confirmed by Akeel and Abd ElFattah (2023) [12]. It had 22 items that were split into five categories: treating subordinates consistently while permitting individualization (5 items); balancing self-centredness and other-centeredness (5 items); retaining autonomy while allowing decision-making authority (4 items); maintaining both proximity and distance (4 items); and enforcing work requirements while allowing for flexibility (4 items). The responses of the subjects were rated and scored using a five-point Likert scale, which goes from zero to five: highly (5), a lot (4), very (3), somewhat (2), barely (1), and not at all (zero). Higher scores on the scale items indicate stronger judgments of paradoxical leadership. The Paradoxical Leadership Scale had a Cronbach’s Alpha Coefficient of 0.99 [12]. In the current study, the Cronbach’s alpha coefficient of the Paradoxical Leadership Scale is 0.93. The cut-off points used to categorize the study variables and their subscales were based on a tertile classification method, which divides the total possible scores into three equal parts: Low: ≤ 33.3% of the total score, Moderate: > 33.3% to ≤ 66.6%, and High: > 66.6%. These cut-offs were applied consistently to the total scores and subscale scores to classify the levels of perception or experience accordingly [30].

The **Paradoxical Leadership Scale (PLS)** was developed by **Zhang et al. (2015)** to assess paradoxical leader behaviors in people management. The original validation involved **five independent samples** and used both **Exploratory Factor Analysis (EFA) and Confirmatory Factor Analysis (CFA)** to establish the scale’s factor structure and validity. EFA initially revealed five factors with nine cross-loading items, which were refined through expert review and further analysis. A second EFA, conducted on a revised 26-item version, led to the removal of four additional items, resulting in a final **five-factor model** that explained **65.6% of the total variance**, with eigenvalues ranging from **1.20 to 6.44**. CFA confirmed the **five-factor structure**, demonstrating strong model fit indices (**CFI > 0.90**,** RMSEA < 0.08**), supporting the **construct validity** of the scale. Additionally, the PLS showed strong **criterion validity**, correlating positively with leadership effectiveness measures such as **employee engagement**,** role proficiency**,** and adaptability**. The reliability of the scale was consistently high, with **Cronbach’s alpha exceeding 0.90**, indicating strong internal consistency.

### Career maturity questionnaire

This scale was approved by Abd El-Hamid et al. (2024) [13] after being taken from Ismail (2022) [16]. to evaluate nurses’ levels of professional maturity. The twenty items were broken down into four dimensions, each of which had five items: vocational self-concept, career exploration, career planning, and career decision-making. Employing a five-point Likert scale, where one represents strongly disagree and five represents strongly agree, nurses’ answers were evaluated. Nurses who score higher on the scale items are perceived as having more career maturity. Internal consistency and tool reliability were assessed using Cronbach’s alpha coefficient, which was found to be high (0.79) [13]. In the current study, the Cronbach’s alpha coefficient of the Career Maturity Questionnaire is 0.83. The cut-off points used to categorize the study variables and their subscales were based on a tertile classification method, which divides the total possible scores into three equal parts: Low: ≤ 33.3% of the total score, Moderate: > 33.3% to ≤ 66.6%, and High: > 66.6%. These cut-offs were applied consistently to the total scores and subscale scores to classify the levels of perception or experience accordingly [30].

The **Career Maturity Questionnaire** was developed and validated by **Ismail et al. (2022)** to assess vocational high school students’ career maturity. The original validation process included **Exploratory Factor Analysis (EFA)**, which identified a three-factor structure comprising **Career Planning and Self-Concept**,** Career Decision-Making**,** and Career Exploration**. The Kaiser-Meyer-Olkin (KMO) index was **0.908**, indicating strong sampling adequacy, and Bartlett’s test of sphericity was significant (***p*** < **0.001**), confirming the suitability of factor analysis. Parallel analysis further validated the three-factor structure, with all factor loadings exceeding **0.4**, indicating strong construct validity. Internal consistency was assessed using **McDonald’s Omega (0.831)**,** Cronbach’s Alpha (0.830)**,** and Guttman’s Lambda-2 (0.834)**, demonstrating high reliability. The scale showed improved psychometric properties compared to previous career maturity instruments, making it a valid and reliable tool for assessing career development.

### The organizational learning scale (OLS)

(Nafei, 2016b) [24] created this scale, and Atalla et al., 2022 [31] verified it. This 25-item measure is divided into five primary subscales: knowledge management (six items), employee empowerment (five items), organizational conversion (five items), dynamics of learning (five items), and technology application (four items). These items must be rated by respondents using a 5-point Likert scale, where 1 represents never and 5 represents always. The nurses’ perspective of organizational learning increases with the scale items’ scores. The Cronbach’s alpha coefficient, which measured the tool’s internal consistency and turned out to be high at 0.90, was used to evaluate its reliability [31]. In the current study, the Cronbach’s alpha coefficient of the Organizational Learning Scale is 0.79. The cut-off points used to categorize the study variables and their subscales were based on a tertile classification method, which divides the total possible scores into three equal parts: Low: ≤ 33.3% of the total score, Moderate: > 33.3% to ≤ 66.6%, and High: > 66.6%. These cut-offs were applied consistently to the total scores and subscale scores to classify the levels of perception or experience accordingly [30].

The **Organizational Learning Scale (OLS)** was originally developed by **Nafei (2016b)** and later validated by **Lyman et al. (2022) and Atalla et al. (2022)** to assess organizational learning in various workplace settings, including healthcare. The scale underwent rigorous psychometric testing, including **Exploratory Factor Analysis (EFA) and Confirmatory Factor Analysis (CFA)**, to establish its factor structure and construct validity. EFA confirmed a **five-factor structure** comprising **dynamics of learning**,** organizational conversion**,** employee empowerment**,** knowledge management**,** and technology application**, with all factor loadings above **0.40**. CFA demonstrated strong model fit indices (**CFI > 0.90**,** RMSEA < 0.08**), confirming the robustness of the scale. The OLS also exhibited **criterion validity**, as it significantly correlated with related constructs such as **employee engagement**,** innovation**,** and organizational performance**. Internal consistency was high, with **Cronbach’s alpha reported at 0.90**, indicating excellent reliability. The scale was further validated in a healthcare context by **Atalla et al. (2022)**, ensuring its applicability for assessing organizational learning among nurses.

### Tools validity

To guarantee language and conceptual equivalency, the research tools were translated after a thorough validation procedure. First, bilingual professionals with experience in healthcare and organizational research independently translated the Paradoxical Leadership Scale (PLS), Career Maturity Questionnaire, and Organizational Learning Scale (OLS) from English into Arabic. To find any differences, a different group of multilingual experts then translated the text back into English. After comparing the back-translated versions with the originals, changes were made to preserve semantic accuracy. The final Arabic translations were examined for clarity, applicability, and cultural fit in an Egyptian healthcare setting by a team of five subject matter experts.

The organizational learning scale, career maturity questionnaire, and paradoxical leadership scale were subjected to confirmatory factor analysis to guarantee accuracy. The Bartlett Test of Sphericity and the Kaiser-Meyer-Olkin (KMO) were the first instruments used to evaluate sample adequacy. A significance criterion of 0.05 and a minimum KMO value of 0.60 are required for the Bartlett Test of Sphericity.

According to the data, the career maturity scale had a value of 0.928 (P 0.000), the organizational learning scale had a value of 0.911 (P 0.000), and the paradoxical leadership scale had a value of 0.889 (P 0.000). Factor loadings for all concepts examined in this study were higher than the suggested threshold of 0.70 [32], and convergent validity is satisfied based on the average variance extracted (AVE) values for each research variable dimension [33]. The average variance extracted (AVE) values for each component were used to evaluate convergent validity. Convergent validity is indicated by AVE values larger than 0.50, which show that the construct accounts for the bulk of the variance. By comparing the squared correlations between the constructs with the AVE values, discriminant validity was evaluated. Since each AVE value was above the squared correlations, discriminant validity was deemed to be satisfied. As a result, the measures employed in this investigation were found to possess both discriminant and convergent validity.

### Ethical considerations

The study procedure was accepted by the College of Nursing’s Damanhour University Research Ethics Committee, and the research code is (104-f). Before signing their consent, nurses were informed of the study’s goal, and each questionnaire was assigned a code number to safeguard their privacy and identification. In addition to being given the choice to leave the study, the researchers were guaranteed that the data would only be utilized for research.

Through several safeguards, the study guaranteed participants’ confidentiality and anonymity. Responses were coded using participant ID numbers instead of names, and nurses’ identifiers were removed before data analysis. After being fully informed about the goal of the study and being made aware of their right to withdraw at any time without consequences, all participants gave their informed consent. Data was safely secured, with electronic files password-protected to avoid unwanted access, and paper-based documents kept in a locked cabinet. Throughout the research process, participant data were handled ethically, and privacy was maintained by adhering to ethical guidelines, such as the Declaration of Helsinki.

### Pilot study and reliability

10% of the nurses (*n* = 38) approved the pilot project to safeguard the tools’ usefulness and usability and to spot any possible problems or obstacles during data collection. There was nothing to be altered. Pilot trial participants were prohibited from continuing the study to avoid data contamination. The findings showed that no changes were required because every item was clear, pertinent, and in line with the goals of the study. There was no uncertainty or difficulty in understanding the questions, according to the participants. Strong internal consistency was confirmed by the pilot study’s Cronbach’s alpha values for reliability, which matched those of the full study. The scales successfully captured the intended constructs, as evidenced by the adequate variation in response patterns and the lack of floor or ceiling effects. These results confirmed that the equipment was appropriate for collecting large amounts of data.

### Overcame the problem of common method biases

The authors used a combination of statistical, procedural, and design controls to overcome common method bias (CMB). To lessen social desirability bias and promote truthful responses, they guaranteed participant secrecy and anonymity. To reduce consistent response patterns, they also divided the questionnaire into several measures, used a variety of response styles, and gave clear instructions. The conclusion that no single factor explained the majority of the variance was confirmed by Harman’s single-factor test, indicating that CMB was not a serious worry. Additionally, the measurement model was confirmed by confirmatory factor analysis (CFA), which guaranteed separate and uncorrelated components. The authors also performed a pilot study with 10% of the sample to improve clarity and remove any ambiguities that can cause CMB. By combining these techniques, they successfully reduced any possible CMB effects in the study.

### Data collection

Participants received individualized copies of the study questionnaires. Each nurse received a questionnaire by hand from the researchers, who then gathered the completed forms. Each nurse had two minutes to explain the purpose of the study before being asked to return it to the researcher. To guarantee the respondents’ objectivity, the integrity of their ideas, and the completion of all questions, these scales were filled out in front of the researcher. It was simple to keep an eye on the delivery and collect data to ensure the best response rate because they were linked to specific working units. As a token of appreciation for their participation, participants received small snacks.

15 to 20 min should be enough time to finish the questions. Two months, from September 2024 to November 2024, were used to collect the data. All of the nurses’ inquiries were answered, and explanations were offered.

### Data analysis

To guarantee accuracy and dependability, the study used a standardized data coding and entry process. SPSS software was used to enter and analyze the data, and categorical variables were recoded as needed. Responses were double-checked for accuracy before analysis to reduce mistakes, and a data-cleaning process was carried out, which included checking for missing values and using imputation methods as needed. To guarantee data integrity, outliers were also investigated. These procedures improved the dataset’s quality and made sure that clean, well-structured data was used for statistical analyses, which increased the study’s validity and dependability.

IBM SPSS AMOS (Version 23) and IBM SPSS Statistics (Version 23) were used to analyze the data. Frequencies and percentages were used to describe the demographic characteristics of the individuals, and mean and standard deviations were used to determine the three main study variables: organizational learning, career maturity, and paradoxical leadership. They also used an independent sample t-test and a one-way analysis of variance to see if the research variable changed based on demographic characteristics. The relationship between the primary research variables was ascertained through the use of Pearson’s correlation analysis. A regression study revealed a direct relationship between professional maturity and paradoxical leadership. Cronbach’s alpha and composite reliability (CR) were used in the study to confirm that the scale items were legitimate. To further guarantee the accuracy of the study’s components, several confirmatory factor analyses were carried out.

The indirect effect of paradoxical leadership on nurses’ career maturity as mediated by organizational learning was investigated using a structural equation model. key statistical terms used in Structural Equation Modeling (SEM) are clarified to better interpret the study’s findings and understand how organizational learning mediates the relationship between paradoxical leadership and career maturity as follows: The path coefficient is a standardized value that represents the strength and direction of the relationship between two variables in a model. It functions like a regression coefficient, indicating how much a change in one variable affects another. A higher absolute value suggests a stronger relationship. Indirect Effect is the effect of an independent variable on a dependent variable through a mediator. It is calculated by multiplying the path coefficients of the two direct relationships involved. In this study, paradoxical leadership influences career maturity indirectly via organizational learning. Model Fit Indices are statistical values used to evaluate how well the proposed model aligns with the observed data. A good model fit suggests that the relationships between variables accurately represent real-world interactions.

- **Chi-square per degree of freedom (X²/df)** – A measure of how well the model fits the data, where lower values indicate a better fit.
- **Comparative Fit Index (CFI) & Incremental Fit Index (IFI)** – Both compare the model’s fit to a baseline model; values closer to **1.000** suggest a perfect fit.
- **Root Mean Square Error of Approximation (RMSEA)** – Estimates the error in model approximation, with values below **0.08** indicating an acceptable fit.

## Results

The study findings indicate that 59.5% of nurses are under 30 years of age (mean = 30.92 ± 10.41 years), and the majority are female (75.3%). Most nurses (66.6%) hold a bachelor’s degree, and 55.5% are married. In terms of clinical placement, nurses are distributed across internal medicine (15.0%), surgical departments (21.6%), and critical care units (23.7%). Regarding experience, 43.7% have less than five years of total work experience (mean = 9.11 ± 6.51 years), while 60.3% have worked in their current unit for less than five years (mean = 6.07 ± 5.12 years). In terms of training, 80.0%, 73.9%, and 77.1% of nurses reported not having received prior training in organizational learning, career maturity, and paradoxical leadership, respectively. Nonetheless, a high level of interest was expressed, with 88.9%, 91.3%, and 90.5% of nurses indicating a desire to receive training in those respective areas (Table 1).

**Table 1:** Distribution of the studied nurses according to demographic data (*n* = 380)

Demographic characteristics	No.	%
**Age (years)**		
< 30	226	59.5
30 –<40	125	32.9
40 –<50	25	6.6
≥ 50	4	1.1
**Mean ± SD**	**30.92 ± 10.41**	
**Sex**		
Male	94	24.7
Female	286	75.3
**Qualification**		
High school diploma	25	6.6
Nursing institute diploma	78	20.5
Bachelor of nursing	253	66.6
Masters	18	4.7
PhD	6	1.6
**Current working unit**		
Surgery	82	21.6
ER	90	23.7
Internal	57	15.0
Other	151	39.7
**Marital status**		
Single	154	40.5
Married	211	55.5
Widowed	9	2.4
Divorced	6	1.6
**Experience years of nursing**		
< 5	166	43.7
5-<10	121	31.8
10-<15	59	15.5
15-<20	18	4.7
≥ 20	16	4.2
**Mean ± SD**	**9.11 ± 6.51**	
**Experience years of current unit**		
< 5	229	60.3
5-<10	79	20.8
10-<15	54	14.2
15-<20	10	2.6
≥ 20	8	2.1
**Mean ± SD**	**6.07 ± 5.12**	
**Attend training on PLS**		
Yes	76	20.0
No	304	80.0
**Like to attend training on PLS**		
Yes	338	88.9
No	42	11.1
**Attending OLS training**		
Yes	99	26.1
No	281	73.9
**Like to attend OLS training**		
Yes	347	91.3
No	33	8.7
**Attending career maturity training**		
Yes	87	22.9
No	293	77.1
**Like to attend career maturity training**		
Yes	344	90.5
No	36	9.5

### Perceived level of paradoxical leadership, career maturity, and organizational learning among nurses

65.0% of nurses reported that their superiors exhibit a high degree of contradictory leadership, according to the research. Additionally, 57.1% of nurses showed a high level of organizational learning, while 78.4% of nurses displayed a high degree of career maturity. Organizational learning obtained the highest mean score (Mean ± SD = 96.71 ± 19.11) among the factors that were measured, indicating its importance in nursing workforce professional growth (Table 2).

**Table 2:** Distribution of the Paradoxical leadership, career maturity, and organizational learning among nurses according to their levels and mean percent score (*n* = 380)

	Low		Moderate		High		Total score	Mean score	Mean percent score
	No.	%	No.	%	No.	%	Mean ± SD	Mean ± SD	Mean ± SD
**Paradoxical Leadership Scale (PLS)**	**2**	**0.5**	**131**	**34.5**	**247**	**65.0**	**80.10 ± 17.14**	**3.64 ± 0.78**	**72.82 ± 15.58**
Treating subordinates uniformly while allowing individualization	12	3.2	105	27.6	263	69.2	18.94 ± 4.76	3.79 ± 0.95	75.78 ± 19.03
Combining self-centeredness with other-centeredness	16	4.2	106	27.9	258	67.9	18.57 ± 4.87	3.71 ± 0.97	74.26 ± 19.48
Maintaining decision control while allowing autonomy	10	2.6	122	32.1	248	65.3	15.08 ± 3.84	3.77 ± 0.96	75.38 ± 19.22
Enforcing work requirements while allowing flexibility	29	7.6	189	49.7	162	42.6	12.85 ± 3.98	3.21 ± 1.0	64.24 ± 19.92
Maintaining both distance and closeness	8	2.1	140	36.8	232	61.1	14.67 ± 3.74	3.67 ± 0.93	73.33 ± 18.68
**Organizational Learning Scale (OLS)**	**6**	**1.6**	**157**	**41.3**	**217**	**57.1**	**96.71 ± 19.11**	**3.87 ± 0.76**	**71.71 ± 19.11**
Dynamics of learning	6	1.6	125	32.9	249	65.5	20.0 ± 3.95	4.0 ± 0.79	75.0 ± 19.73
Conversion of the organization	4	1.1	136	35.8	240	63.2	19.92 ± 3.90	3.98 ± 0.78	74.61 ± 19.50
Employee empowerment	12	3.2	161	42.4	207	54.5	19.09 ± 4.14	3.82 ± 0.83	70.46 ± 20.72
Knowledge management	18	4.7	148	38.9	214	56.3	22.72 ± 5.20	3.79 ± 0.87	69.68 ± 21.68
Application of technology	24	6.3	148	38.9	208	54.7	14.97 ± 3.60	3.74 ± 0.90	68.57 ± 22.52
**Nurses Career Maturity Questionnaire**	**2**	**0.5**	**80**	**21.1**	**298**	**78.4**	**82.33 ± 12.46**	**4.12 ± 0.62**	**77.91 ± 15.58**
Career exploration	2	0.5	80	21.1	298	78.4	20.79 ± 3.29	4.16 ± 0.66	78.93 ± 16.46
Career planning	6	1.6	99	26.1	275	72.4	20.33 ± 3.44	4.07 ± 0.69	76.63 ± 17.18
Vocational self-concept	4	1.1	82	21.6	294	77.4	20.68 ± 3.36	4.14 ± 0.67	78.42 ± 16.81
Career decision making	6	1.6	84	22.1	290	76.3	20.53 ± 3.48	4.11 ± 0.70	77.64 ± 17.39

### Relationship among Paradoxical leadership, career maturity, and organizational learning among nurses

The correlation matrix evaluating the connections among organizational learning, career maturity, and paradoxical leadership, based on a sample of 380 nurses, showed that all of the links were statistically significant at *p* = 0.05. Organizational learning (*r* = 0.136) and career maturity (*r* = 0.169) were positively connected with paradoxical leadership, suggesting that nurses’ organizational learning and career maturity tend to rise as they perceive more paradoxical leadership. Additionally, a strong association (*r* = 0.200) was discovered between career maturity and organizational learning, indicating that these two factors usually develop together and support one another in nurses’ professional development (Table 3).

**Table 3:**
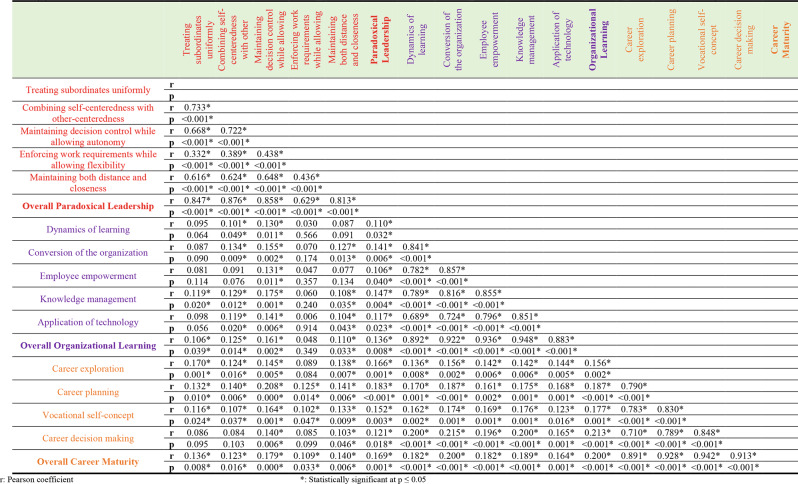
Correlation between the Paradoxical leadership, career maturity, and organizational learning among nurses (*n* = 380)

**Fig. 3:**
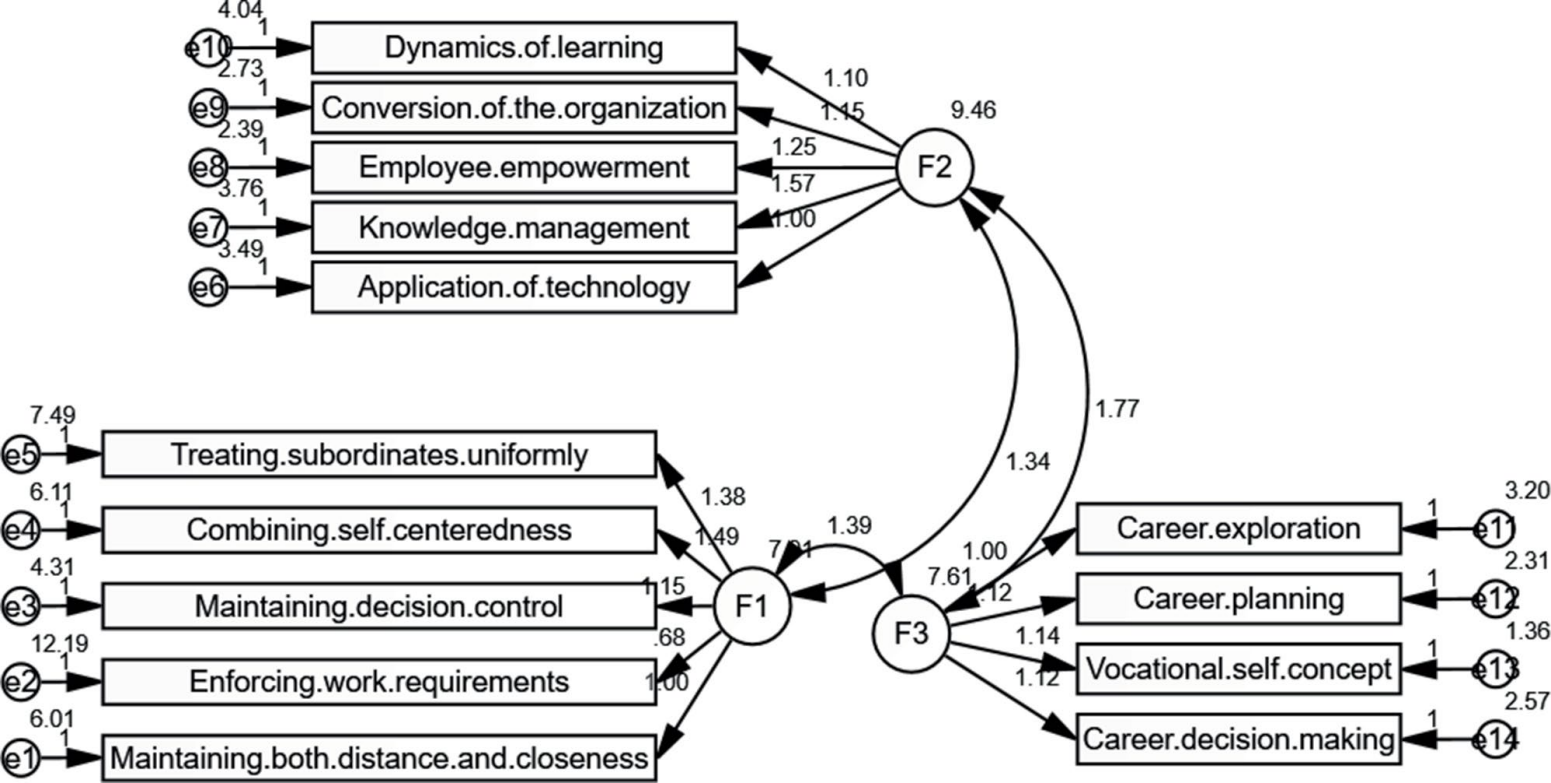
Confirmatory Factor Analysis (CFA) model illustrating the factor structure of paradoxical leadership, organizational learning, and career maturity scales. F1: Paradoxical Leadership Scale (PLS) F2: Organizational Learning Scale (OLS) F3: Nurses Career Maturity Questionnaire

A Confirmatory Factor Analysis model examining the connections among three important latent components is shown in Fig. 3. The Paradoxical Leadership Scale (PLS), represented by Factor 1 (F1), is made up of observed indications such as treating subordinates consistently and enforcing work requirements. These indicators indicate a leader’s capacity to control seemingly incongruous behaviors. The Organizational Learning Scale (OLS) is embodied in Factor 2 (F2), which reflects the organization’s ability to expand and adapt through variables including learning dynamics and technology application. Lastly, the model suggests that paradoxical leadership (F1) and organizational learning (F2) have both direct and indirect effects on nurses’ career maturity, which is represented by Factor 3 (F3). This is determined by a particular questionnaire that includes career exploration and vocational self-concept.

### Standardized regression coefficient weights among Paradoxical leadership, career maturity, and organizational learning among nurses, with the mediating role of organizational learning

**Fig. 4:**
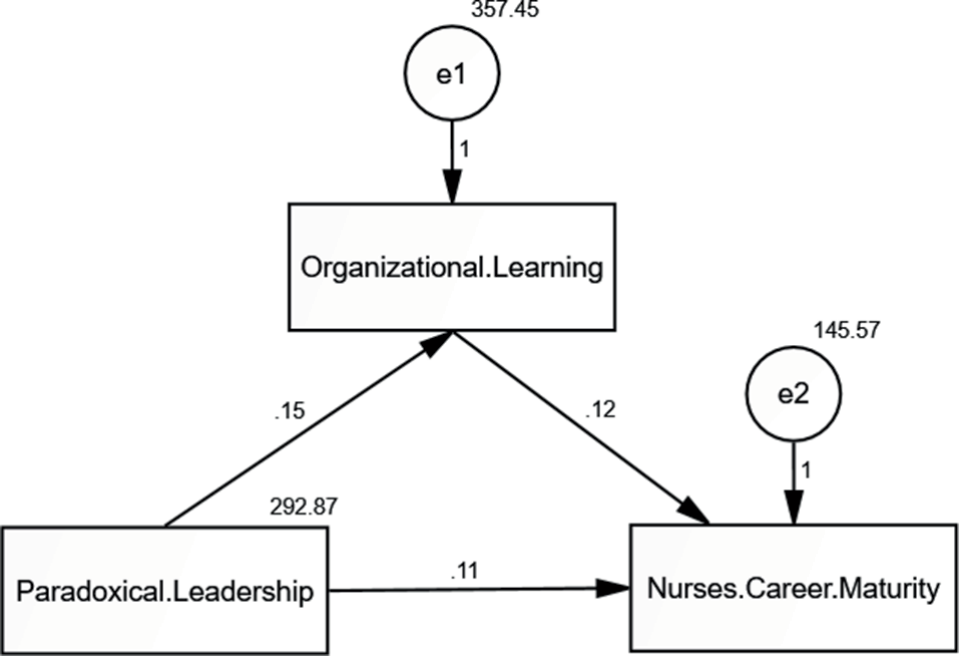
Path analysis of the direct and indirect effect of Paradoxical Leadership on Nurses’ Career Maturity mediated by Organizational Learning

With an emphasis on the mediating function of organizational learning, this study investigates the direct and indirect effects of paradoxical leadership, organizational learning, and career maturity among nurses. The findings indicate that organizational learning is strongly impacted by paradoxical leadership (standardized path coefficient = 0.136, *p* = 0.007), and career maturity is positively impacted by organizational learning (standardized path coefficient = 0.180, *p* < 0.001). Career maturity is likewise directly impacted by paradoxical leadership (standardized path coefficient = 0.144, *p* = 0.004). With an approximate 0.756 standardized unit increase in career maturity for every unit increase in paradoxical leadership, the indirect effect of paradoxical leadership on career maturity via organizational learning is calculated to be 0.0245 (0.136 × 0.180), indicating that organizational learning partially mediates this relationship. With a Root Mean Square Error of Approximation (RMSEA) of 0.073, an Incremental Fit Index (IFI) and Comparative Fit Index (CFI) of 1.000, and a chi-square value per degree of freedom of 10.260/3, model fit indices further verify the model’s adequacy. These results highlight how organizational learning plays a critical mediating role in the relationship between paradoxical leadership and nurses’ career maturity (see Table 4; Fig. 4).

**Table 4:** The direct and indirect effect of Paradoxical leadership on nurses’ career maturity mediating by organizational learning

			Direct effect	Indirect effect	Estimate	S.E.	C.*R*.	*P*
Organizational Learning	<---	Paradoxical Leadership	0.152		**0.136**	0.057	2.679*	0.007*
Nurses Career Maturity	<---	Paradoxical Leadership	0.105	0.018	0.144	0.037	2.874*	0.004*
Nurses Career Maturity	<---	Organizational Learning	0.118		**0.180**	0.033	3.591*	< 0.001*

Model fit parameters CFI; IFI; RMSEA (1.000; 1.000; 0.073)

Model χ^2^/df. 10.260/3 *p* ≤ 0.001

CFI: Comparative Fit Index, IFI: Incremental Fit Index, RMSEA: Root Mean Square Error of Approximation

## Discussion

Many people believe that paradoxical leadership is a good way to manage the complicated situations that modern organizations encounter [3]. In terms of their work attitudes, involvement, dedication, and exceptional performance, nurses benefit from paradoxical leadership [11]. The development of nurses is ironically aided by leadership, which offers them more chances to achieve professional success and maturity [3].

### Perceived level of paradoxical leadership, career maturity, and organizational learning among nurses

Most nurses believed their supervisors had a high level of paradoxical leadership, according to the study’s findings. For a variety of reasons, the majority of nurses thought their managers exhibited a high degree of paradoxical leadership. To ensure both protocol adherence and patient care adaptability, executives frequently strike a balance between authority and flexibility. Secondly, the collectivist culture encourages leaders to combine strict decision-making with sympathetic engagement by fostering collaboration and mutual support. Third, supervisors may need to strike a balance between control and empowerment to promote both discipline and innovation due to the dynamic nature of nursing work, which contains unforeseen challenges. Furthermore, hospital leaders may be able to embrace both tradition and change through organizational learning practices, which would reinforce paradoxical leadership characteristics. Finally, hospital leadership training programs may potentially have an impact on nurses’ attitudes by emphasizing balancing stability with adaptability to enhance overall team performance.

This result is consistent with (Yang et al., 2021) [34], (Sparr, et al., 2022) [35], (He and Yun, 2022) [36], (Stynen and Semeijn, 2023) [37], (Chen and Yang, 2023) [38], (Akeel and Abd ElFattah, 2023) [30] who found that a high overall degree of perception about paradoxical leadership behaviors was held by more than half (55.6%) of the nurses in the study.

Additionally, this study found that the majority of nurses believe they are at a high level of career maturity. Several reasons contribute to this. Professional growth is fostered by the challenging nature of the nursing profession, which calls for constant skill improvement, critical thinking, and adaptability. Second, nurses’ perception of career maturity is enhanced by the unambiguous professional development opportunities, mentorship programs, and training that are offered by healthcare organizations’ established career routes. Third, nurses frequently get a great deal of practical experience in managing a variety of patient cases, which improves their decision-making skills and confidence in their positions. Furthermore, leadership support and organizational learning may foster an atmosphere that promotes resilience, self-analysis, and ongoing development. Lastly, nurses’ views of their careers may be further supported by cultural values that emphasize dedication, responsibility, and professional commitment in the healthcare industry.

This finding is reliable with those of Seo and Kim (2019) [39], Dodd (2019) [40], (Abdul Rahim, et al., 2021) [41], and Abd El-Hamid, et al., (2024) [13] who revealed that a high degree of career maturity was had by the majority of head nurses (87.5%). On the other hand, this study’s findings contradict those of other investigations (Hamzah, et al., 2020) [42], (Sunzi, et al., 2023) [43], and (Rayan and Abd Rabou, 2023) [44], who claimed that nurses had a moderate degree of career maturity.

In addition, the study’s findings showed that the majority of nurses think highly of organizational learning. There are some reasons why the majority of nurses have positive opinions of organizational learning, according to the study. First of all, the study hospital is a knowledge-intensive setting where enhancing patient care and keeping up with medical developments require ongoing education. Also, the collaborative nature of nursing work fosters a culture of shared learning, wherein seasoned nurses mentor newer staff, reinforcing organizational learning. organized training programs, workshops, and professional development initiatives give nurses the chance to improve their skills and knowledge. Additionally, nurses’ favorable opinions of learning chances at work might be influenced by leadership support for innovation and evidence-based practice. Finally, the culture of learning is probably strengthened by organizational policies that encourage feedback, reflection, and continual improvement, making it a valued part of their work experience.

This study supports the findings of Elhoseney et al. (2020) [45], El Desoky (2021) [46], and Ahmed et al. (2023) [47], who discovered that 68.0% of staff nurses thought their organization had a strong organizational learning culture. The findings of this study, however, conflict with those of (Moafimadani et al. 2020) [48], (Akhtar et al., 2021) [49], and (Atalla et al., 2022) [31], who stated that the nurses in the study thought they experienced a modest amount of organizational learning in general.

The study’s findings showed that paradoxical leadership and professional maturity were connected. There are many explanations for the study’s findings that paradoxical leadership and professional maturity are related. First, paradoxical leaders strike a balance between empowerment and authority, enabling nurses to grow independently while upholding professional norms. **Second, such leadership develops critical thinking and adaptation, which are essential elements of career maturity, by promoting both flexibility and discipline. Third, paradoxical leadership fosters psychological safety, which allows nurses to ask for feedback, grow from their mistakes, and keep improving. Additionally, nurses are better able to negotiate complex healthcare contexts and advance their careers when leaders can manage paradoxes, such as stability and change or control and freedom. In the end, this leadership approach strengthens the connection between paradoxical leadership and career maturity by promoting both career progress and confidence.

This result aligns with research conducted by Zhang et al. (2015) [3], Derksen et al. (2017) [11], Zhang et al. (2022) [12], and Li et al. (2023) [50], which found that paradoxical leaders allow nurses to demonstrate more competent, flexible, and proactive behaviors while also increasing the sustainability of their careers.

### Standardized regression coefficient weights among Paradoxical leadership, career maturity, and organizational learning among nurses with the mediating role of organizational learning

The results of this study also demonstrated that paradoxical leadership and career maturity were mediated by organizational learning. The study’s findings highlighted the critical role that organizational learning plays in professional growth by showing that organizational learning mediates the association between paradoxical leadership and career maturity. First, paradoxical leaders encourage nurses to embrace continuous learning while upholding professional discipline by striking a balance between rigidity and flexibility, thereby creating a learning-oriented environment. Second, nurses can learn, apply, and improve their knowledge through organizational learning, which enhances their ability to make decisions and advance their careers. Third, nurses gain more self-assurance and become more prepared for career progression when they are assisted in learning from their experiences and overcoming obstacles. Furthermore, paradoxical leadership results in real career advancement since a robust learning culture reinforces leadership influence. As a result, organizational learning functions as a bridge, converting leadership behaviors into improved nursing career maturity.

The study’s results are consistent with those of Zhang, et al., (2015) [3], (Derksen, et al., 2017) [11], (Zhang et al. 2022) [12], and (Li, et al., 2023) [50], (Ishtiaq, et al., 2024) [51], and (Atalla et al. 2024) [52], who stated that paradoxical leadership promotes nurses’ opportunity for organizational learning, demonstrates concern for their professional growth, and gives them increasing chances to achieve professional success and maturity.

Likewise, The study discovered a positive association between paradoxical leadership, career maturity, and organizational learning. Career maturity was directly impacted by organizational learning functioning as a mediator. To encourage nurses to embrace both innovation and adherence to professional norms, paradoxical leadership first cultivates a learning culture. Second, nurses gain the critical thinking, problem-solving, and adaptability necessary for career maturity through opportunities for continual learning. Third, nurses who participate in organizational learning develop their confidence, hone their skills, and become more self-reliant in making decisions, all of which have a direct impact on their professional development. Furthermore, learning experiences are improved by leadership that encourages both structure and flexibility, guaranteeing that nurses convert information into professional maturity. In the end, organizational learning is an important mechanism that reinforces the connection between professional maturity and paradoxical leadership.

This finding is compatible with (Zhang, et al., 2015) [3], (Rahman, et al., 2016) [19], (Derksen, et al., 2017) [11], (Naeem and Iqbal, 2022) [53], (Anyienwe, 2022) [20], (Zhang et al. 2022) [12], (Li, et al., 2023) [50], and (Ishtiaq, et al., 2024) [51], and (Atalla et al. 2024) [54], who stated that paradoxical leadership fosters organizational learning culture, which is a major factor in nurses’ professional maturity and a precursor to career advancement.

## Conclusion

This study demonstrates the important connections among nurses’ career maturity, organizational learning, and paradoxical leadership, highlighting the pivotal function of organizational learning as a mediator. According to the research, nurses’ professional development and preparedness for the workforce can be improved by cultivating a culture that values learning. Hospital administrators and nursing managers should put in place organized leadership training programs that include paradoxical leadership principles to put these insights into reality. This will assist nurse leaders in striking a balance between flexibility and control. Furthermore, mentorship programs should be set up to assist with career development by matching seasoned nurses with less experienced employees to promote professional development and knowledge exchange.

To ensure that aspiring nurse leaders are prepared to handle challenging workplace situations, healthcare organizations should incorporate paradoxical leadership training into their nurse leadership development programs. To improve nurses’ career maturity and adaptability in changing healthcare environments, legislators should also support programs for continuous organizational learning, such as frequent workshops, training in evidence-based practice, and interdisciplinary collaboration. Healthcare businesses may cultivate a nursing workforce that is resilient, knowledgeable, and prepared by putting these strategic strategies into practice.

### Strengths and limitations

This study adds to a better knowledge of how leadership styles affect nursing practice by providing insightful information about the connection between organizational learning, career maturity, and paradoxical leadership. Context-specific insights from the study of nurses in Egypt can guide regional healthcare leadership initiatives. The study’s design, which takes organizational learning into account as a mediating factor, adds depth and a fresh viewpoint to the corpus of information on nursing leadership. The research emphasizes the value of balanced leadership and ongoing professional development in fostering nurses’ career advancement and organizational success by looking at these factors collectively.

The limitations include the challenge of establishing causal relationships between organizational learning, career maturity, and paradoxical leadership due to the study’s cross-sectional design. Furthermore, the results may not be fully generalizable to other healthcare settings in Egypt or around the world because the study was carried out in a single hospital. Additional possible drawbacks include self-reported data bias, since personal perceptions or social desirability may have an impact on participants’ answers. Additionally, the results could have been affected by uncontrolled confounding variables, such as hospital rules, workplace culture, or individual motivations.

### Implications for nursing practice

The study’s conclusions have important ramifications for nursing practice especially when it comes to creating an atmosphere that encourages nurses to advance in their careers. By striking a balance between conflicting expectations, such as fostering personal development and advancing organizational objectives, paradoxical leadership can enable nurses to successfully handle challenging situations in their work. Nursing leaders can foster an atmosphere where nurses feel encouraged to grow in their careers by implementing this leadership style. This method fosters critical thinking, adaptability, and resilience—qualities that are crucial for professional advancement in the rapidly changing healthcare industry. Higher career maturity among empowered nurses increases the likelihood that they will provide high-quality care, show leadership potential, and participate in ongoing professional development [55].

The study also emphasizes the role that organizational learning plays as a mediator between career maturity and paradoxical leadership. Hospitals and healthcare institutions can improve nurses’ abilities, expertise, and self-assurance by cultivating a culture of lifelong learning. This focus on education gives nurses the skills they need to remain current with evidence-based practices and adapt to changes in healthcare practices. Additionally, job satisfaction, retention rates, and general nursing competency can all be enhanced by a robust organizational learning framework. To support career maturity and overall nursing performance, nursing leaders should prioritize both leadership development and the creation of strong educational programs. This will benefit the individual nurse as well as the healthcare organization overall. These findings highlight how crucial it is to promote self-efficacy to strengthen EBP implementation and advance the nursing profession. The nursing profession stands out for its human-centered approach and ethical dedication to patient care. Nursing leaders should foster a culture of support, continuous education, and communication to empower nurses. Encouraging active organizational learning in opportunities improves patient outcomes and nurses’ career maturity [56].

### Future studies

Future research should examine how paradoxical leadership affects nursing career maturity over the long term in various healthcare environments, taking into account cultural and organizational differences. Furthermore, studies could look at the precise processes by which organizational learning affects nurses’ professional growth, like determining the best learning tools or techniques. It would also be beneficial to look at how paradoxical leadership compares to other leadership philosophies, such as transformational or servant leadership, in terms of developing professional maturity. Lastly, to obtain a more thorough grasp of how organizational learning and leadership affect nursing competency and job satisfaction, future research might involve a greater number of nurses in the sample with a range of specializations and career stages.

## Data Availability

Upon a reasonable request, the relevant author will make the datasets created and examined during the current work available.
